# Peer mentoring for eating disorders: results from the evaluation of a pilot program

**DOI:** 10.1186/s40337-019-0245-3

**Published:** 2019-06-03

**Authors:** Jennifer Beveridge, Andrea Phillipou, Zoe Jenkins, Richard Newton, Leah Brennan, Freya Hanly, Benjamin Torrens-Witherow, Narelle Warren, Kelly Edwards, David Castle

**Affiliations:** 1 Eating Disorders Victoria, Melbourne, VIC Australia; 2 Centre for Mental Health, Swinburne University of Technology, PO Box 218, Hawthorn, VIC 3122 Australia; 3 Department of Psychiatry, St Vincent’s Hospital, Melbourne, VIC Australia; 4 Department of Psychiatry, The University of Melbourne, Melbourne, VIC Australia; 5 Department of Mental Health, Austin Health, Melbourne, VIC Australia; 6 Peninsula Mental Health Service, Peninsula Health, Melbourne, VIC Australia; 7 Department of Psychiatry, Monash University, Melbourne, VIC Australia; 8 School of Behavioural and Health Science, Australian Catholic University, Melbourne, VIC Australia; 9 Centre for Eating, Weight and Body Image, Melbourne, VIC Australia; 10 School of Social Sciences, Monash University, Melbourne, VIC Australia

**Keywords:** Eating disorders, Peer mentoring, Quality of life, Treatment, Peer work

## Abstract

**Background:**

Eating disorders (EDs) are serious psychiatric illnesses that have high rates of morbidity and mortality, and low long-term recovery rates. Peer mentor programs (PMPs) have been associated with reduced psychiatric hospitalisation and shorter lengths of stay for those with other severe mental illnesses. The present study evaluated the feasibility and preliminary efficacy of a PMP for individuals with EDs in improving symptomatology and quality of life.

**Methods:**

Thirty mentees and seventeen mentors were recruited. The PMP involved thirteen sessions over 6 months. Participants completed measures assessing ED symptomatology, quality of life (QoL), mood and perceived disability. Changes in symptomatology before and after the PMP were tested by Wilcoxon signed rank tests. Semi-structured interviews were conducted for qualitative evaluation of the PMP.

**Results:**

The program was deemed to have moderate feasibility with eight of 30 mentees, and two of 17 mentors withdrawing. Completion rates ranged from 2 to 16 sessions, and between 3 and 45 weeks. Mentees demonstrated improvements in body mass index, QoL, ED symptomatology, mood (depression, anxiety and tension/stress) and perceived disability from pre- to post-program. Mentors demonstrated significant increases in ED symptomatology, but no worsening of QoL, mood or perceived disability. Qualitative findings from both mentees and mentors were positive: emergent themes included hope for recovery, a sense of agency and inspiration gained from interaction with someone with lived experience of an ED.

**Conclusions:**

This pilot study suggests feasibility of the PMP for individuals with EDs. Mentees demonstrated improvements in ED symptomatology, QoL, mood and perceived disability. However, the increase in ED symptomatology reported by the mentors over the PMP highlights potential risks and the need for thorough monitoring while preliminary evaluation is undertaken. The mentoring relationship was a positive experience for both mentees and mentors, instilling an increased hope for recovery in mentees and an opportunity for mentors to reflect on their own recovery with increased confidence. The novel relationship formed throughout mentorship highlights a potential gap in current clinical support services, which warrants further exploration within a controlled trial.

**Trial registration:**

Australian and New Zealand Clinical Trials Registration Number: ACTRN12617001412325. Retrospectively registered: 05/10/2017. Date of first enrolment: 20/01/2017. https://www.anzctr.org.au/Trial/Registration/TrialReview.aspx?id=373741&isReview=true

**Electronic supplementary material:**

The online version of this article (10.1186/s40337-019-0245-3) contains supplementary material, which is available to authorized users.

## Plain English summary

Individuals recovered from an eating disorder (‘mentors’) and individuals currently with an eating disorder (‘mentees’) participated in a peer mentoring program. Thirteen mentoring sessions occurred over 6 months. Participants were assessed on key measures relating to their eating disorder and overall wellbeing, at the beginning and end of the program. Mentees overall showed improvements in eating disorder symptoms. A few mentors showed a small increase in eating disorder symptoms over the course of the program, but no worsening in quality of life or depression/anxiety. Interviews with mentors and mentees found many positive aspects relating to the experience of participation, and highlighted some areas that could be refined in future studies. Overall, the results are encouraging, but further research is required to extend and expand our knowledge about the exact place peer support has in the treatment journey of people with an eating disorder.

## Background

Eating disorders are serious psychiatric conditions with high rates of morbidity, mortality and associated high economic costs [1]. Anorexia nervosa (AN) is arguably the most dramatically life-shortening eating disorder, with medical morbidity and mortality consequent upon starvation, and suicide also being common amongst those with the illness [2, 3]. AN can be particularly difficult to treat given individual engagement in treatment is often complicated by the lack of acceptance of the need for help by the patient [4, 5]. In this context, the importance of social support and encouragement regarding treatment is a key component of care (e.g. [6, 7]).

Despite recognition of the importance of social support, little research attention has been given to incorporating peer support into treatment plans for people with eating disorders. Peer support engages people with their own ‘lived experience’ of illness in helping others [8]. Davidson et al. [9] have recently reviewed the literature on peer support in people with a severe mental illness and articulated the elements that characterise their work, namely instillation of hope through positive self-disclosure, role modelling and building a relationship based on trust, acceptance and empathy. Peer mentor programs have been associated with reduced psychiatric hospitalisation and shorter lengths of stay in a sample of patients with a diagnosis of a psychotic disorder, bipolar disorder or major depressive disorder [6]. Despite these positive indicators, little evidence is yet available in the literature to support peer mentoring in eating disorder populations.

In their systematic review of peer support in eating disorders, Fogarty and colleagues [10] identified four studies (*N* = 270) of particular relevance. One of these studies, a mentoring program for women with eating disorders, found quality of life and adherence to treatment were enhanced in participants matched with a peer mentor (*n* = 58) vs. those unmatched (*n* = 49) [7]. A separate eight-week mentoring pilot program engaged adolescent girls (*N* = 31) with subclinical eating disorders and reported a significant reduction in disordered eating resultant from working with a peer mentor [11]. Two more recent studies add to this literature. Ramjan and colleagues [12] performed a proof of concept study exploring the impact of mentoring support in 10 females with an eating disorder and found improvements in hope for recovery in terms of social relationships, family life, romantic relationships and work. The same researchers [13] also describe a 13-week mentoring support program in 11 women with AN (five mentors and six mentees). All but one of the pairs completed the program but there were no significant improvements on qualitative outcomes: this might in part reflect the low number of participants and the fact that not all of them completed the follow-up questionnaires.

In terms of qualitative outcomes, Lippi et al. [14] investigated the themes arising from a six-week mentoring program that matched females (*N* = 6) in the early stages of recovery from an eating disorder with mentors (*N* = 6) in the later stages of recover. The authors reported heightened self-awareness and understanding of the recovery process in mentees post program. Furthermore, mentees reported that they felt better understood, even ‘normal’, consequent of their having been afforded the opportunity to share their experiences with someone who had been through and negotiated issues similar to what they were dealing with. A further eight-week mentoring program addressed body image anxiety in adolescent girls (*N* = 25), yet reported no significant benefits for mentees [15]. In integrating the main findings from the studies included in their review, Fogarty and colleagues [10] underlined the importance of the supportive relationship which developed between mentors and mentees, allowing the mentees a sense of belonging. Ramjan et al. [13], in their study of 11 women with AN (see above) found five key themes emerged form qualitative enquiry, namely: 1) mentees feeling understood; 2) reconnection with the world; 3) mentors’ altruism and self-discovery; 4) hope related to recovery; and 5) the importance of effective communication.

It has been noted in the literature that peer workers can be both positively and negatively affected by providing peer support. In the eating disorder literature, mentors have reported consolidation of skills acquired in recovery and recognition of their own success in recovery [7]. However, some mentors have been found to overinvest emotionally in their mentees’ recovery or succumb themselves to vulnerabilities associated with their previous diagnosis [16]. The more substantial literature on peer mentoring within the wider mental health population suggests that, through facilitating the recovery of others, mentors gain an increased sense of interpersonal competence which validates their own recovery journey [17] and a sense of personal freedom and integrity [18].

The primary aim of the current study was to determine the feasibility and acceptability of a peer mentor program for people with an eating disorder. Secondary aims were to build on the peer mentoring in eating disorder literature by considering whether such an intervention could improve eating disorder symptoms, negative mood and quality of life among mentees. The impact of the program on mentors was also assessed as a tertiary aim. It was hypothesised that participation in the peer mentor program would have high acceptability, improve eating disorder symptomatology and reduce post-specialist treatment weight loss or readmission in the mentees.

## Methods

The current study evaluated a pilot feasibility trial of a peer mentor program (PMP) conducted at Eating Disorders Victoria (EDV), The Melbourne Clinic (TMC), and the Body Image and Eating Disorders Treatment and Recovery Service (BETRS) at the Austin Hospital (inpatient unit) and St. Vincent’s Hospital (intensive day patient program), all in Melbourne, Victoria, Australia. The design and procedure of the study and PMP have been described in detail [19]; brief details have been provided here. The study was granted independent ethics approval by the Human Research Ethics committees at St Vincent’s Hospital, Austin Health and The Melbourne Clinic. Written informed consent was obtained from all participants.

### Participants

Inclusion criteria for mentees were: (1) Current diagnosis of an eating disorder according to the Diagnostic and Statistical Manual of Mental Disorders (DSM-5); (2) transitioning out of an inpatient program or transitioning in/out of an outpatient/day patient program and; (3) currently actively receiving treatment for their eating disorder for the duration of the program.

Inclusion criteria for mentors were: (1) Recovery from an eating disorder for a minimum of one year (note that Beveridge et al. (2018) [10] states that recovery would be sustained for a minimum of two years, but was modified for feasibility); (2) successful recruitment as a staff member at EDV, including referee checks and agreement code of conduct. Mentors were paid employees of EDV, employed specifically for their mentoring role.

There were no exclusion criteria for mentees or mentors. Diagnoses for both mentees and mentors were self-declared.

### Matching process

Mentees and mentors were matched based on information provided by each participant. For mentees, this information included a statement of their values and interests, and preferences for characteristics of their mentor: age, gender, eating disorder history and location. Mentors provided a similar statement of their values and interests, and preferred work style [19]. All participants were matched with at least two of their preferences being met.

### Peer Mentor program

The conceptual model developed for the peer mentor program derived from an integrative peer model [9] and consisted of 13 mentoring sessions of up to 3 hours, every 2 weeks. Activities undertaken were based on peer support and included providing information, emotional support and sharing of personal recovery experiences. An individualised Wellness Plan (see Additional file 1) [19] was developed by the mentee during the first session. Each plan included short term goals in the following domains: living circumstances and skills, health, self-care, social relationships and connectedness, creative interests and hobbies, work/career and education, identity and sense of self, and community roles and responsibilities. The Wellness Plan detailed current positive strategies (e.g., thoughts, feelings, behaviours, characteristics, personal qualities, interests, activities, relationships) that support the mentee in their recovery, as well as highlight any symptoms that indicate the need for additional treatment or professional support.

Mentees and mentors worked together in subsequent sessions towards achieving the identified goals. Activities varied for each mentee and included daily living tasks, making connections with the community, and/or practising social interactions that included food and eating. Throughout the sessions, mentors shared relevant aspects of their own recovery stories to provide hope and empathic support for the challenges experienced by the mentees.

The final session involved completion of a Program Summary that measured the outcomes achieved against the mentees Wellness Plan. Mentees identified strategies and supports to utilise after the completion of the mentoring relationship as they sustained the progress achieved and sought to continue their recovery. At the end of each session, mentors completed an online questionnaire to document mentee progress and to monitor any risks. This information was then reviewed by the EDV program coordinator and follow up occurred with any mentee or mentor who needed additional support.

Mentees were afforded the opportunity to take part in bimonthly group sessions facilitated by two professional staff, including a psychologist. The sessions aimed to increase a sense of belonging during the program, which would extend to an alumni group, and offer a forum to provide feedback on their experience of the program.

Mentors also attended separate bimonthly group supervision sessions facilitated by an eating disorder clinician: attendance was an expectation of their employment. The purpose of the sessions was to provide mentors with case discussion, training and peer support. All mentors attended at least one session, with an average attendance of 72% by mentors across all sessions. In addition, all mentors undertook 3 days of training prior to commencing with the program. The content of the training included orientation to the organisation, the peer work model, safe storytelling, boundaries and expectations, and eating disorder education. Suicide prevention training was provided by an accredited external provider. The training was interactive with case studies, scenarios and reflection.

### Evaluation procedures

Baseline and end-point quantitative assessments were administered before the first mentoring session and following the last mentoring session, respectively. Quantitative assessments included:

i) The Eating Disorders Examination Questionnaire (EDE-Q) [20], a 28-item self-report measure of psychological constructs shown to be clinically relevant in individuals with eating disorders. The EDE-Q has four clinically derived subscales (Restraint, Eating Concern, Weight Concern and Shape Concern) that are used to calculate the Global score, with higher scores representing higher eating disorder symptomatology, with good concurrent validity and acceptable criterion validity [21]. A Global score of 2.3 or higher has been demonstrated to have a positive predictive value (PPV) of 0.56, specificity of 0.96 and sensitivity of 0.83 in determining the presence of clinically significant disordered eating [21].
ii) The Depression Anxiety Stress Scale (DASS-21) [22] is a 21-item self-report instrument designed to measure the three related negative emotional states of Depression, Anxiety and Stress. Recommended cut-off scores indicative of moderate severity for Depression, Anxiety and Stress are ≥13, 9 and 18, respectively [23]. Internal consistency of the DASS subscales is high, with reported Cronbach’s alphas of 0.94, 0.88, and 0.93 for Depression, Anxiety, and Stress respectively [24].
iii) The Brief Disability Questionnaire (BDQ) [25] is a self-report measure of disability in everyday activities and yields subscales of Physical, Mental and Functional Disability, with higher scores indicative of greater disability.
iv) The Assessment of Quality of Life (AQoL-8D) [26] is a psychometric measure that produces scores of eight dimensions of health related quality of life, with an increased score indicative of a greater quality of life. Subscales include Independent Living, Happiness, Mental Health, Coping, Relationships, Self Worth, Pain and Senses.

Basic clinical history and demographic information (e.g. age, gender, eating disorder history) was also collected at baseline, as well as self-reported hospital admission rates at end-point.

Qualitative data were also collected. Mentees and mentors completed three online reflection exercises: one after being matched (baseline), a second at the midpoint of the mentoring sessions, and a third following the last mentoring session. In addition, 12 mentors and 14 mentees took part in recorded qualitative interviews in person or over the phone following completion of the program. These interviews were transcribed verbatim and subjected to thematic analysis [27] and data were collected until saturation was reached [28]. Data were initially coded by two researchers individually using NVivio software and then cross-member checked. Incongruences in coding were discussed between the two researchers until agreement was reached that each code was a valid representation of the research participants’ experiences.

## Results

Thirty mentees (two male) consented to participate in the study. Of these 30 participants, 12 were recruited from the Inpatient Service at the Austin Hospital, 14 from the Day Patient Program at St Vincent’s Hospital (the Body Image & Eating Disorders Treatment & Recovery Service; BETRS), and four from The Melbourne Clinic Inpatient Service. The recruitment period spanned from January 2017 to April 2018.

Mentees were aged 18–50 years (M = 27.8, SD = 9.5); 27 were born in Australia, and one each in South Africa, England and New Zealand. At baseline, six mentees were employed full-time, five employed part-time or casual, eleven were students, seven were unemployed, and one reported their employment status as ‘other’. Highest education status achieved were reported as tertiary by thirteen participants, secondary by seven, vocational course/diploma by three, and five as currently studying (two unreported).

Primary eating disorder diagnosis for mentees was predominantly AN (*N* = 28); one participant reported bulimia nervosa (BN) as their primary diagnosis and one, other specific feeding or eating disorder (OSFED). Age of eating disorder onset ranged from eight to 47 years (M = 18.2, SD = 7.0), and the duration of the eating disorder 1–30 years (M = 8.4, SD = 8.6).

Of the 30 participants enrolled in the study, eight withdrew during the program. Reasons for participant withdrawal included overseas travel, moving interstate, returning home to a regional area after treatment and lack of motivation to engage with the program. Twenty-two participants who did not withdraw from the program were analysed. Of those mentees who completed the study, three completed the 13 sessions in 6 months; the remaining participants completed varying numbers of program sessions (ranging from 2 to 16 sessions) (M = 9.13, SD = 4.22) within varying timeframes (ranging from 3 to 45 weeks) (M = 20.70, SD = 13.24 weeks). While the program was designed as 13 sessions over 6 months, decisions were at times made to facilitate participant inclusion in the program which resulted in some flexibility being tolerated. Not all participants were able to complete 13 sessions in some cohorts due to the program and funding constraints, and some instances when the 6 month period was extended due to circumstances such as participant availability to meet.

Seventeen mentors were employed for the purpose of this program, but two withdrew due to overseas travel and pregnancy. Data from the remaining 15 mentors was analysed. Mentors included fourteen females and one male aged between 23 to 39 years (M = 29.5, SD = 4.3). One mentor was born in England, two in India, and the remaining mentors were all born in Australia. Employment status was recorded as full-time for three mentors, part-time for four mentors, and ‘other’ for two mentors; six mentors reported were students. Six mentors had completed tertiary education, two had completed vocational course/diploma and one had completed secondary school (six unreported).

All mentors had previously been diagnosed with an eating disorder. Primary diagnoses included AN (*n* = 10), BN (*n* = 3), binge-eating disorder (BED) (n = 1) and eating disorder not otherwise specified (EDNOS) (n = 1). The duration of recovery ranged from one to 7.5 years (M = 3.5, SD = 2.1). The age of eating disorder onset ranged from 13 to 25 years of age (M = 17.25, SD = 3.8), whilst the duration of the eating disorder before recovery ranged from two to 15 years (M = 6.25, SD = 3.5).

**Fig. 1:**
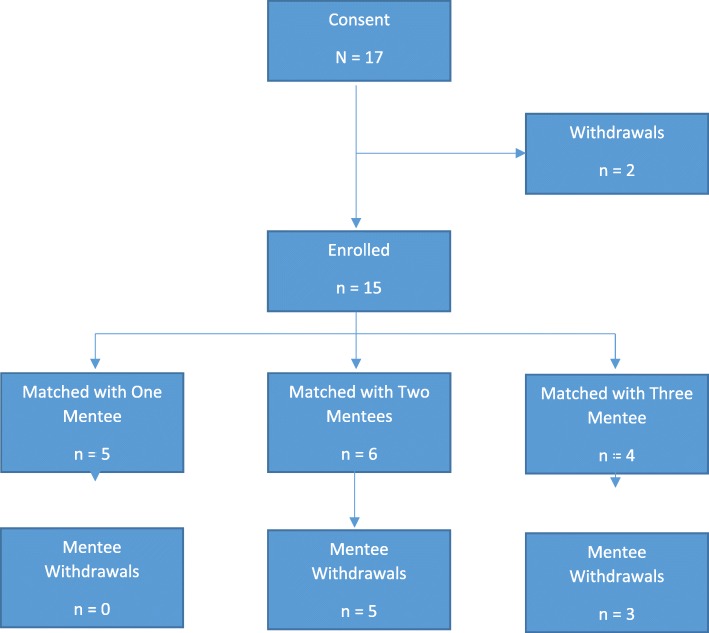
Mentor participation, and number of associated mentee matches and withdrawals. *Note:* In addition, one mentee was matched with two different mentors

Mentors were matched to one or more mentees, dependent on the matching process. Five mentors were each matched with one mentee; six mentors were each matched with two mentees; and four mentors were each matched with three mentees. None of the mentors who were matched with one mentee had any withdrawals; three mentors who were matched with two mentees had one withdrawal each; one mentor who was matched with two mentees had two withdrawals; and three mentors who were matched with three mentees had one withdrawal each (see Fig. 1). All mentees were matched with one mentor, except one. This mentee was matched with two mentors due to first mentor stepping up into the program coordinator role and therefore unable to continue as a mentor.

### Quantitative outcomes: mentees

Eight (36%) of the 22 mentees who completed the program were re-admitted to inpatient psychiatric service or re-admitted to day-patient program during the program, three of whom had multiple admissions. Non-parametric tests (Wilcoxon) were undertaken between baseline and end-point as data were not normally distributed (see Table 1). Overall, mentees were found to have a significant increase in body mass index and also recorded significantly reduced scores on the Restraint, Eating Concern, Shape Concern and Global subscale scores of the EDE-Q, as well as on the Depression, Anxiety and Stress subscales of the DASS. Improvements were found on the BDQ Physical, Mental and Functional Disability subscales. Improved quality of life was also found for the AQoL overall; specifically on the happiness, mental health, relationships and self-worth subscales.

**Table 1:** Clinical and Psychological Characteristics of Mentees^a^

	Baseline M (SD)	Endpoint M (SD)	*Z*	*p*
BMI	17.0 (2.2)	17.7 (3.3)	−2.4	0.02
EDE-Q Restraint	2.6 (1.4)	2.1 (1.4)	−2.3	0.02
EDE-Q Eating Concern	3.0 (1.4)	2.4 (1.5)	−2.5	0.01
EDE-Q Shape Concern	4.2 (1.4)	3.3 (1.6)	−2.9	0.01
EDE-Q Weight Concern	3.6 (1.7)	3.1 (1.8)	−1.9	0.06
EDE-Q Global	3.3 (1.4)	2.7 (1.5)	−2.5	0.01
DASS Depression	19.7 (13.1)	15.5 (13.5)	−2.5	0.01
DASS Anxiety	16.8 (12.0)	12.5 (10.5)	−3.1	0.01
DASS Stress	22.2 (11.2)	18.7 (10.5)	−2.3	0.02
BDQ Physical	6.5 (4.3)	5.1 (4.6)	−2.4	0.02
BDQ Mental	3.5 (1.7)	2.6 (2.0)	−2.4	0.02
BDQ Functional	9.0 (17.5)	4.8 (8.1)	−2.2	0.03
AQoL Individual Living	78.3 (17.5)	82.6 (18.6)	−1.9	0.06
AQoL Happiness	42.7 (17.9)	51.4 (23.5)	−2.5	0.01
AQoL Mental Health	48.5 (18.3)	54.8 (21.6)	−3.0	0.01
AQoL Coping	42.0 (21.7)	49.2 (24.4)	−2.0	0.05
AQoL Relationships	57.8 (16.0)	66.7 (19.5)	−2.4	0.02
AQoL Self-Worth	27.9 (24.2)	40.9 (27.9)	−2.8	0.01
AQoL Pain	81.7 (21.2)	81.8 (21.7)	−0.5	0.65
AQoL Senses	86.0 (12.8)	87.4 (12.2)	−0.9	0.38
AQoL Total	57.1 (15.1)	64.3 (17.4)	−3.1	0.01

^a^Data analysis conducted on 22 mentees who did not withdraw – i.e. those who did not complete all 13 sessions or did not complete within 6 months are still included in the analysis; AQoL scores are standardised unweighted scores

### Quantitative outcomes: mentors

No hospital admissions were reported for any mentor during their mentoring relationships. Non-parametric tests (Wilcoxon) were undertaken between baseline and end-point as the data were not normally distributed (see Table 2). Mentors demonstrated an increase in eating disorder symptoms, specifically in the Eating Concern, Weight Concern and Global subscales of the EDE-Q: no absolute scores were in the pathological range at any time-point. No significant change was recorded in measures of mood (depression, anxiety, stress), disability or quality of life.

**Table 2:** Clinical and Psychological Characteristics of Mentors^a^

	Baseline M (SD)	Endpoint M (SD)	*Z*	*p*
EDE-Q Restraint	0.1 (0.2)	0.1(.15)	−0.1	0.91
EDE-Q Eating Concern	0.05 (0.1)	0.2 (0.3)	−2.0	0.04
EDE-Q Shape Concern	0.6 (0.5)	0.7 (0.6)	−1.4	0.18
EDE-Q Weight Concern	0.6 (0.7)	0.9 (0.8)	−2.1	0.04
EDE-Q Global	0.3 (0.4)	0.5 (0.4)	−2.3	0.02
DASS Depression	2.1 (2.7)	2.1 (2.1)	−0.3	0.73
DASS Anxiety	1.5 (1.9)	2.0 (3.2)	−0.6	0.52
DASS Stress	6.8 (6.1)	8.8 (7.1)	−1.4	0.16
BDQ Physical	0.5 (1.0)	1.9 (3.9)	−1.2	0.24
BDQ Mental	0.1 (0.5)	0.3 (0.9)	−0.8	0.41
BDQ Functional	0.9 (1.4)	0.5 (1.2)	−1.3	0.19
AQoL Individual Living	96.7 (6.9)	93.0 (12.5)	−0.8	0.42
AQoL Happiness	79.6 (10.2)	78.8 (10.0)	−0.2	0.81
AQoL Mental Health	79.4 (8.9)	80.6 (15.4)	−0.1	0.97
AQoL Coping	75.6 (12.8)	74.4 (13.2)	−1.1	0.28
AQoL Relationships	87.9 (9.0)	88.1 (9.3)	−0.1	0.91
AQoL Self-Worth	80.0 (12.1)	81.7 (11.0)	−0.8	0.40
AQoL Pain	86.7 (19.9)	82.0 (26.8)	−1.1	0.25
AQoL Senses	91.3 (7.6)	89.7 (9.0)	−0.9	0.38
AQoL Total	84.6 (7.8)	83.9 (9.0)	−0.2	0.83

^a^Data analysis completed on 15 mentors who were matched a mentee – no data were collected on mentors not matched with a mentee; data were only collected for the first mentoring relationship – i.e. if the mentor was matched more than one mentee, only the first mentoring relationship has been recorded; AQoL scores are standardised unweighted scores

### Qualitative data: mentees

**Fig. 2:**
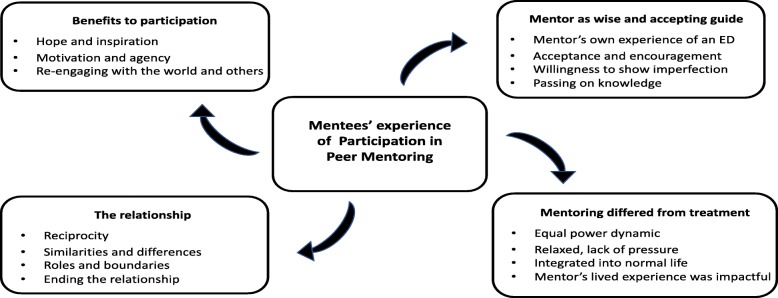
Themes and subthemes from mentee experiences

We provide here a synopsis of the qualitative outcomes. A full exposition will be the subjects of a separate publication. Four themes relating to mentees’ experiences of participating in the peer mentoring program emerged from thematic analysis, and are presented with their subthemes in Fig. 2 and examples of quotes pertinent to the themes, presented in Table 3.

**Table 3:** Example Quotes from Qualitative Themes

Themes	Example Quotes
Mentees	
1. Benefits to Participation	*I think it was just inspiring … actually this is a life that maybe I do want more than I want to be sick … I’ve had an eating disorder for thirteen years and this is the first time that I’ve had even an inkling that I’d even possibly want to get better*. -Michelle
2. Mentor as Wise and Accepting Guide	*It’s just so nice to talk to someone who wasn’t going to judge you, and just accepted you had a bad week, that’s ok, you know, keep moving forward, keep going. -*Rene
3. The Relationship	*It’s a two way street thing […]I did find that aspect more comforting to deal with I guess, […*] *not feeling like centre stage and … we did share a lot of personal things. –*Jackie
4. Mentoring Differed from Treatment	*It felt a lot more relaxed, more like a friendship than a professional relationship, but it still was in a sense, but … we were able to work on things like eating out, and doing nice things, as well as [talking about issues] at the same time. -*Bailey
Mentors	
1. Connection with Self and Others	*Before you even go through the door you know you’ve got something in common, there’s a shared connection there […*] *and I have found that there’s a lot more ease when that connection is already there. -*Sandy
2. Exploration of Mentors’ Own Recovery	*[Recovery] was not a fun time by any means, […*] *but then to be able to make even a tiny of a fraction of change to make that a little bit easier for someone else, just makes it just so incredibly worthwhile … It [reinforces] everything that you learn in recovery.* -Steph
3. Support is Essential in the Face of Self-Doubt	*Some of [the] sessions were quite raw, but [EDV] were always there … for me to talk to … that had a wonderful effect*. -Sandy
4. Reciprocal Gains are Possible	*I got such joy seeing that actually I was helping somebody else […*] *and I think it does become this cyclic thing that the more you give the more you have to give*. -Kim
5. Role Clarity and Boundary Management	*Extracting yourself from a pseudo-friendship is hard, especially when there’s been a genuine connection. There is the risk/fear of making the participant feel personally rejected if you […*] *don’t continue a relationship post-mentoring.* -Rory

*Perceived benefits* of participation were threefold. The most frequently cited was enhanced hope for recovery. This in turn increased motivation and agency towards recovery goals. Mentoring also assisted mentees to re-engage with others and the ‘outside world’, following their isolation experience resulting from the illness. The theme, *Mentor as a wise and accepting guide*, depicts the significance of mentors for mentees’ recovery. Mentees appreciated the wisdom that was delivered in a spirit of non-judgmental and unconditional encouragement. Importantly, mentees’ receptivity to this knowledge was at least partly contingent on mentors’ similar experiences of an ED, and their willingness to share their vulnerabilities and imperfections. *The relationship* theme highlights the importance of the quality of the relationship. Reciprocity and clear boundaries were considered essential. The few mentees who reported a lack of mutual connection and understanding in the relationship were the same few who found the program less helpful in general, suggesting the centrality of the mentoring relationship to the program’s success. The experience of ending the relationship due to program completion was the most frequently cited challenge to participation, and was characterised by sadness, followed by acceptance. All participants described mentoring as distinctly *different from clinical treatment*, in the sense that it was more collaborative, equal, and relaxed. It was also appreciated for being more integrated into ‘normal life’, and more impactful due to the mentor’s lived experience of an eating disorder.

### Qualitative data: mentors

**Fig. 3:**
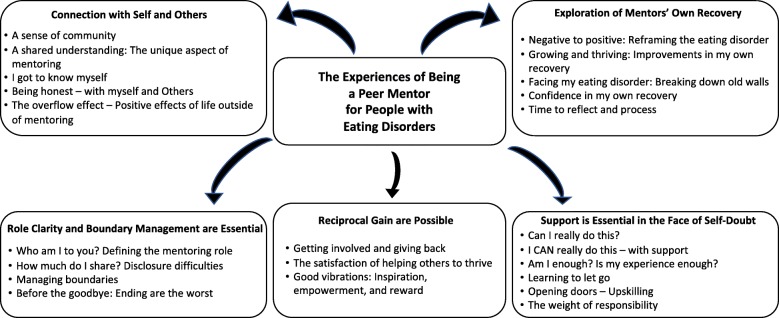
Themes and subthemes from mentor experiences

Qualitative analysis of the mentors revealed five key themes, each with a number of subthemes (see Fig. 3).

The first theme captured the *sense of connection with self and others* within the eating disorder community, with mentors emphasising a unique sense of community as one of the cornerstones of the positive mentoring experience. The second theme, the recurring sense that mentors were able to *explore and gain perspective on their own recovery* through participation the program, was driven by the mentors’ reflection on the opportunity to reframe their ED and recovery experience from one which was predominantly negative, into something which could benefit others. The third theme captured the common reports of self-doubt by mentors in the early stages of the program, followed by an *increase in confidence* as the program went on, which was largely attributed to the training, supervision, and support provided. The fourth theme detailed the hopes for and *reasons behind commencing* the mentoring role, and the often-surprising *gains* reported. These gains included the development of personal and professional skills, as well as the empowerment, inspiration, and satisfaction that came with supporting a peer. The fifth and final theme captured some of the most commonly identified *challenges* of the program for mentors: delineating the mentor role, managing disclosure, and defining appropriate boundaries. Ongoing supervision and support were seen as essential in pre-empting and managing these challenges.

## Discussion

We report here on the feasibility, acceptability and quantitative and qualitative outcomes associated with participation in a peer mentoring program for individuals with an eating disorder (mostly AN). Feasibility was established in that recruitment targets were met, albeit retention rates were somewhat lower than anticipated. In future studies, retention rates might be enhanced through closer attention to supporting the mentor-mentee dyad and ensuring a suitable ‘fit’ between the two. Also, as the field advances a more informed approach will be possible regarding which sort of patient will most benefit, and at what illness stage; the same issues pertain to mentors. Nicholls and colleagues [29] for example, used a participatory action research paradigm in an mentor/mentee program with women with AN, paying close attention to individual needs and views in an iterative manner.

Over the time-course of the program, the mentees recorded significant positive changes in terms of BMI, In contrast to many consumer’s experience of discharge being a time for weight loss. This is a critically important clinical outcome. The mentee rehospitalisation rate of eight participants is difficult to interpret without an adequate control group. It is also unclear whether rehospitalisation is a measure of successful ongoing engagement with services post discharge and therefore a positive outcome of the peer mentoring experience or reflects a negative deterioration. Whilst statistically significant, many of the changes in EDE Q and DASS were modest in scale. (see Table 1). Having said this, overall EDE-Q score improvements were greater than the 0.45 point change considered ‘clinically significant’ [30]. Overall these results show modest improvement post discharge from intensive treatment which can be considered clinically significant in the context of the high rates of relapse generally seen post discharge., in particular the BMI increase. Longer-term follow-up, beyond the scope of this program, will be required to determine longitudinal trajectories.

Qualitative findings revealed the extent to which the mentees saw the peer worker relationship as different from ‘usual’ clinical interventions in terms of key items such as reciprocity and sharing. Strong themes emerged regarding the instillation of hope for recovery and a sense of agency; inspiration was gleaned from being able to access support from someone who had the lived experience of illness and of recovery.

There is little by way of published research with which to compare our findings.

The fact that mentors on average showed an increase in Weight Concern, Shape Concern and Global subscales of the EDE-Q over the course of the study arguably reflects the fact that they were engaged actively in a therapeutic manner with mentees and were guiding and supporting them on their recovery journey. This may have been associated with mentees reflecting on their own illness experience. Importantly, mentors’ baseline levels of concern regarding weight and eating were low, and none entered the ‘pathological’ range on any item. There was also no deterioration in Depression, Anxiety, Stress or QoL measures.

The qualitative outcomes for mentors in our study were positive about the opportunity to use the ‘negative’ experience of their own illness in a positive manner to help others. This said, it is vital that regular monitoring of the wellbeing of mentors be undertaken as a core part of this sort of work, and sufficient support needs to be provided to ensure no adverse clinical outcomes eventuate.

There are a number of limitations to this study. The number of participants was small, there was some heterogeneity in the clinical diagnoses (albeit most mentees had AN) and the number of sessions conducted and duration of program varied between participants (i.e. only three participants complete 13 sessions in 6 months as planned). Participants were also completing their treatment as usual following transition out of an inpatient service, or transition out of or into a day patient service; therefore, the benefits of the program over treatment as usual remain unclear. A key drawback is the lack of a control condition, but it must be remembered that this was a pilot study of feasibility and acceptability. Given the encouraging outcomes from this study, including a treatment as usual group would be the next step in terms of scientific rigour.

## Conclusions

The current study reports encouraging results from a peer mentor program to support those with eating disorders. After participation in a peer facilitated program, mentees demonstrated improvements in BMI, eating disorder symptomatology, mood, disability and quality of life. However, mentors reported an increase in eating disorder symptomatology over the course of the program; highlighting the need for vigorous mentor support and monitoring in peer support programs for individuals with eating disorders. Qualitative results highlighted that the mentoring relationship was a positive experience for both mentees and mentors, instilling an increased hope for recovery in mentees and an opportunity for mentors to reflect on their own recovery with increased confidence. The novel relationship formed throughout mentorship highlights a potential gap in current clinical support services which warrants further exploration within a controlled trial.

## Additional file

Additional file 1: Wellness plan. (DOCX 38 kb)

## Abbreviations

AN: Anorexia nervosa
AQoL-8D: Assessment of Quality of Life, 8 Dimension Edition
BDQ: Brief Disability Questionnaire
BED: Binge Eating Disorder
BETRS: Body Image and Eating Disorders Treatment and Recovery Service
BN: Bulimia nervosa
DASS: Depression Anxiety Stress Scale
DSM-5: Diagnostic and Statistical Manual of Mental Disorders, 5th Edition
ED: Eating disorder
EDE-Q: Eating Disorder Examination Questionnaire
EDNOS: Eating Disorder Not Otherwise Specified
EDV: Eating Disorders Victoria
OSFED: Other specified feeding and eating disorder
PMP: Peer Mentor Program
QoL: Quality of Life
TMC: The Melbourne Clinic
