# Supplementary material for: Peer mentoring for eating disorders: results from the evaluation of a pilot program
Source: J Eat Disord. 2019 Jun 3;7:13. doi: 10.1186/s40337-019-0245-3 (PMC6545742; doi:10.1186/s40337-019-0245-3)
Supplement: Supplementary file 1 — Wellness plan. (DOCX 38 kb) [file 40337_2019_245_MOESM1_ESM.docx]

**My Wellness Plan**

***My Wellness Toolbox for Self Care***

***What do you do to keep yourself well or to help you feel better?***

Your wellness tools and techniques are things you can do to support your wellness. They might be tools you have learned and are currently using because they work for you, or they might be things you have heard of but not yet tried.

| Wellness tools I currently use | Wellness tools I would like to try |
| --- | --- |
|  |  |

***Daily Maintenance Plan***

***…to keep me feeling healthy and well***

Words that describe me when I feel healthy and well.

……………………………………………………………………………………………………………………………………………………………………………………………………………………………………………………………………………………………………………………………………………………

When I am healthy and well, I…(consider your thoughts, feelings, behaviours, characteristics, personal qualities, interests, activities, relationships, etc).

*e.g. When I am healthy and well, I…like to explore my creative side.*

……………………………………………………………………………………………………………………………………………………………………………………………………………………………………………………………………………………………………………………………………………………………………………………………………………………………………………………………………………………………………………………………………………………………………………………………………………………………………………………………………………………………………………………………………………………

Consider how you need to care for yourself, to keep you feeling healthy and well (consider what you might need to do on a daily basis and also less frequently).

*e.g. To maintain my health and wellness, I need to…spend time connecting with others.*

……………………………………………………………………………………………………………………………………………………………………………………………………………………………………………………………………………………………………………………………………………………………………………………………………………………………………………………………………………………………………………………………………………………………………………………………………………………………………………………………………………………………………………………………………………………

List the things you know are important to do for your health and wellness, however you find it difficult to do them. What makes it difficult?

*It is difficult for me to do X, because*….......................................................

……………………………………………………………………………………………………………………………………………………………………………………………………………………………………………………………………………………………………………………………………………………………………………………………………………………………………………………………………

***Identifying Triggers***

***What is a trigger?*** *A trigger is an external circumstance, situation, cue or event that may impact your level of health and wellness and make you feel uncomfortable (e.g. people, places, certain topics, etc).*

***List your triggers…***

*…………………………………………………………………………………………………………………………………………………………………………………………………………………………………………………………………………………………………………………………………………………………………………………………………………………………………………………………………………………………………………………………………………………………………………………………………………………………………………………………*

***My Trigger Action Plan***

What might I do to avoid or limit potential triggers? If I am triggered, what can I do to prevent me from feeling worse? How can I respond in a helpful way so I do not feel worse?

*……………………………………………………………………………………………………………………………………………………………………………………………………………………………………………………………………………………………………………………………………………………………………………………………………………………………………………………………………………………………………………………………………………………………………………………………………………………………………………………………………………………………………………………………………………………*

***Early Warning Signs***

***Early warning signs*** *are signs of change (e.g. symptoms, thoughts, images, feelings, mood, behaviours) that may indicate a person is starting to feel unwell or starting to feel worse. Identifying these signs will help your Mentor to ‘check in’ with you if things are not going well.*

***What are my early warning signs?***

Signs I have observed in myself? What others have noticed in me? (e.g. sleeping in later each day, or taking a couple of days to return messages from friends)

…………………………………………………………………………………………………………………………………………………………………………………………………………………………………………………………………………………………………………………………………………………………………………………………………………………………………………………………………………………………………………………………………………………………………………………………………………………………………………………………

***My Action Plan…***

What must I do when I recognise early warning signs? What can I do each day until I feel better?

***How can I take action before things get worse?*** (consider your Wellness Toolbox and anything else that might be helpful)

…………………………………………………………………………………………………………………………………………………………………………………………………………………………………………………………………………………………………………………………………………………………………………………………………………………………………………………………………………………………………………………………………………………………………………………………………………………………………………………………

***How do I know if things are breaking down?***

Signs that will indicate my situation is worsening further, becoming more serious and potentially even unsafe for me. This might include symptoms, thoughts, images, feelings, mood, and behaviours (e.g. being unable to leave the house, or not responding to messages from friends).

…………………………………………………………………………………………………………………………………………………………………………………………………………………………………………………………………………………………………………………………………………………………………………………………………………………………………………………………………………………………………………………………………………………………………………………………………………………………………………………………

***My Action Plan…***

At this point, caring for myself is the number one priority.

*What do I need to do now? (e.g. make an immediate appointment with my treating team).*

…………………………………………………………………………………………………………………………………………………………………………………………………………………………………………………………………………………………………………………………………………………………………………………………………………………………………………………………………………………………………………………………………………………………………………………………………………………………………………………………………………………………………………………………………………………………………………………………………………………………………………………………………………………………………………………………………………

***Supports***

*Who can provide me with helpful support? Who can I turn to, to support me if I’m struggling?*

| **Who (name)** | **Our relationship (e.g. friend, professional)** | **How they can support me?** |
| --- | --- | --- |
|  |  |  |
|  |  |  |
|  |  |  |
|  |  |  |

***Values and Goals***

Values are our heart’s deepest desires for how we want to interact with and relate to the world, other people and ourselves. They are a *direction* we keep moving in. Values are different to goals, because they are ongoing, whereas goals are the things we want to *achieve* along the way (Russ Harris, 2010).

***What are my values?*** *What really matters to me deep down? What sort of person do I want to be? (e.g. a caring, supportive friend).*

……………………………………………………………………………………………………………………………………………………………………………………………………………………………………………………………………………………………………………………………………………………………………………………………………………………………………………………………………………………………………………………………………………………………………………………………………………………………………………………………………………………………………………………………………………………

***Goal setting***

***What goals would I like to work towards during the Peer Mentoring Program?*** How can I practice acting on my values in working towards my goals?

For example, you might like to set goals in any of the domains listed below:

- Living circumstances and skills
- Health (physical and emotional/mental health; self-care)
- Social relationships and connectedness
- Creativity/interests/hobbies
- Work/career and/or education
- Identity and sense of self/self-esteem
- Roles and responsibilities
- Other areas?

***My Goals***

- *Consider SMART Goals - Specific, Measurable, Achievable, Relevant, Time-limited*

1. *…………………………………………………………………………………………………………………………………………………….*

*What steps or tasks can my Mentor help me with in working towards this goal?*

1. *…………………………………………………………………………………………………………………………………………………….*
2. *…………………………………………………………………………………………………………………………………………………….*
3. *…………………………………………………………………………………………………………………………………………………….*

| **SMART Goal** | **Tasks/Steps to reach this goal** |
| --- | --- |
| 1. |  |
| 2. |  |
| 3. |  |
| 4. |  |

Adapted from Wellness Recovery Action Plan (WRAP), Mary Ellen Copeland (2002) <http://mentalhealthrecovery.com/>
